# Hairpin RNA Targeting Multiple Viral Genes Confers Strong Resistance to Rice Black-Streaked Dwarf Virus

**DOI:** 10.3390/ijms17050705

**Published:** 2016-05-11

**Authors:** Fangquan Wang, Wenqi Li, Jinyan Zhu, Fangjun Fan, Jun Wang, Weigong Zhong, Ming-Bo Wang, Qing Liu, Qian-Hao Zhu, Tong Zhou, Ying Lan, Yijun Zhou, Jie Yang

**Affiliations:** 1 Institute of Food Crops, Jiangsu Academy of Agricultural Sciences/Nanjing Branch of Chinese National Center for Rice Improvement/Jiangsu High Quality Rice R & D Center, Nanjing 210014, China; qiezi324@163.com (F.W.); liwqtomorrow@163.com (W.L.); zhujinyanrain@163.com (J.Z.); weidadefan@sohu.com (F.F.); wangjunjaas@aliyun.com (J.W.); wgzhong0503@139.com (W.Z.); 2 Jiangsu Co-Innovation Center for Modern Production Technology of Grain Crops, Yangzhou University, Yangzhou 225009, China; 3 CSIRO Agriculture, GPO Box 1600, Canberra, ACT 2601, Australia; ming-bo.wang@csiro.au (M.-B.W.); qing.liu@csiro.au (Q.L.); qianhao.zhu@csiro.au (Q.-H.Z.); 4 Institute of Plant Protection, Jiangsu Academy of Agricultural Sciences, Nanjing 210014, China; zhoutong@jaas.ac.cn (T.Z.); 15005167120@163.com (Y.L.); yjzhou@jaas.ac.cn (Y.Z.)

**Keywords:** RBSDV, hpRNA, RNAi, multiple viral genes, strong resistance

## Abstract

Rice black-streaked dwarf virus (RBSDV) belongs to the genus *Fijivirus* in the family of *Reoviridae* and causes severe yield loss in rice-producing areas in Asia. RNA silencing, as a natural defence mechanism against plant viruses, has been successfully exploited for engineering virus resistance in plants, including rice. In this study, we generated transgenic rice lines harbouring a hairpin RNA (hpRNA) construct targeting four RBSDV genes, *S1*, *S2*, *S6* and *S10*, encoding the RNA-dependent RNA polymerase, the putative core protein, the RNA silencing suppressor and the outer capsid protein, respectively. Both field nursery and artificial inoculation assays of three generations of the transgenic lines showed that they had strong resistance to RBSDV infection. The RBSDV resistance in the segregating transgenic populations correlated perfectly with the presence of the hpRNA transgene. Furthermore, the hpRNA transgene was expressed in the highly resistant transgenic lines, giving rise to abundant levels of 21–24 nt small interfering RNA (siRNA). By small RNA deep sequencing, the RBSDV-resistant transgenic lines detected siRNAs from all four viral gene sequences in the hpRNA transgene, indicating that the whole chimeric fusion sequence can be efficiently processed by Dicer into siRNAs. Taken together, our results suggest that long hpRNA targeting multiple viral genes can be used to generate stable and durable virus resistance in rice, as well as other plant species.

## 1. Introduction

Rice is one of the major cereal crops in the world and is important for global food security. Rice black-streaked dwarf virus disease is a destructive viral disease, caused by rice black-streaked dwarf virus (RBSDV), which mainly infects rice and maize plants in East Asian countries [1,2,3]. This virus disease caused serious yield losses of rice and maize in Japan during the 1960s [4]. In China, epidemic outbreaks of the rice black-streaked dwarf disease usually resulted in a grain yield decrease of 10%–40%, and in some cases, such as in the outbreaks of 1997–1998 and 2007–2009, a total loss of grain production in the rice planting areas of southern China [5]. RBSDV is persistently transmitted by an insect vector, small brown planthopper (SBPH, *Laodelphax striatellus* Fallén), that carries RBSDV after feeding on viruliferous rice or other RBSDV hosts. Unlike the rice stripe virus (RSV) that can be transmitted through SBPH ovary to the offspring [6], RBSDV cannot be transmitted to offspring of SBPH through ovary. Consequently, a disease epidemic by RBSDV depends on the abundance of viruliferous hosts and the number of SBPH carrying RBSDV. These characteristics of RBSDV transmission lead to the difficulty in screening for RBSDV-resistant rice germplasms and evaluating the resistance level of rice materials. Two methods are currently used to assay for RBSDV resistance in rice, namely field RBSDV nursery and artificial inoculation. Results obtained in field RBSDV nursery assays are usually less reliable due to the difficulties in controlling the density and percentage of SBPH carrying RBSDV. In contrast, assay results from the artificial inoculation method are generally reliable, although it is time-consuming and laborious for large-scale screening and evaluation of rice collections [6,7].

The RBSDV genome is 29,141 bp in length and consists of 10 linear dsRNA segments, which are named *S1*–*S10* [8,9]. *S5*, *S7* and *S9* segments each contain two open reading frames (ORF), and the others (*S1*–*S4*, *S6*, *S8* and *S10*) each contain a single ORF [9,10,11,12]. The core particles of RBSDV are composed of four proteins, P1 (RNA-dependent RNA polymerase), P2 (probably a core protein), P3 (putative capping enzyme) and P8 (minor core protein), which are encoded by *S1*, *S2*, *S3* and *S8*, respectively [9,13,14]. The RBSDV particle contains at least two out layer proteins, P4 (B-spike protein) and P10 (major outer capsid protein), which are encoded by *S4* and *S10*, respectively [9,11,15]. P6, P7-1 and P9-1 are three non-structural proteins of the RBSDV particle [16]. P6 functions as an RNA silencing suppressor, which effectively inhibits the RNA silencing process and aggravates the development of the disease symptoms by interaction with P9-1 protein [17,18,19]. P7-2 as a special non-structural protein plays an important role for pathogenicity during the stage of virus infection [13]. The P9-1 protein is the smallest component unit for RBSDV viroplasm formation [20]. P10 protein is an important outer capsid protein, which takes part in the assembly of virus particles. Understanding the biological information of the RBSDV genome is helpful for creating rice germplasm conferring resistance to RBSDV via the RNA silencing-based strategy [21,22].

In the process of long-term interaction with pathogens, plants have evolved a variety of complex and effective resistance mechanisms against virus infection [22]. Virus resistance mediated by natural resistance genes and RNA silencing-mediated virus resistance are currently two major research focuses [23]. An increasing number of research works reported that many virus resistance genes play important roles in restraining viral replication and preventing virus movement in plants. The *N* gene with the conserved TIR-NBS-LRR domain is the first virus resistance gene to be cloned from *Nicotiana tabacum* [24]. A signal defence mechanism is activated to limit the virus infection after the TIR domain of the N protein recognizes and interacts with the coiled-coil domain P50 of the tobacco mosaic virus (TMV) replication enzyme [25]. Tomato Tm-2^2^ and sw-5 antiviral proteins, both containing a conserved CC-NBS-LRR domain, function in resistance to tomato mosaic virus (ToMV) and tomato spotted wilt virus (TSWV), respectively [26,27]. However, cloning and characterization of viral resistance genes are very difficult in rice germplasms because of the lack of germplasm resources with a high level of resistance to virus. At present, only one gene (*STV11-R*) and five quantitative trait loci (QTLs; *Stv-bi*, *qSTV11^IR24^*, *qSTV11^TQ^*, *qSTV11^KAS^* and *qSTV11^SG^*) conferring resistance to rice stripe virus (RSV) have been cloned and/or characterized [28,29]. However, no RBSDV-resistant germplasm has been discovered so far amongst rice collections [22]. An increasing number of studies indicates that using the RNA silencing-based strategy to generate virus-resistant rice germplasm resources is highly promising [30].

RNA silencing is an evolutionarily-conserved natural defence mechanism against viruses in a diversity of plants [21]. In plant antiviral RNA silencing, double-stranded (ds) RNA of viral sequences is processed by Dicer-like (DCL) proteins into 21–24 nt small interfering RNA (siRNA), which forms an RNA-induced silencing complex (RISC) with an Argonaute protein and guides RISC to bind and cleave single-stranded viral RNA [31]. RNA silencing has been exploited for genetic engineering of virus resistance in plants. The efficiency and specificity of the RNA silencing-based technology in developing virus-resistant plants has been well established [21,32]. Transgenic rice expressing siRNA-generating hairpin RNA (hpRNA) designed based on a specific sequence of viral gene has been shown to inhibit viral RNA accumulation, hence replication and infection of the viruses [16,33]. Transgenic rice harbouring an hpRNA vector targeting *pC3* and *pC4* genes showed an enhanced resistance to RSV [34]. Pns6-(viroplasm associated protein and movement protein), P8-(major outer capsid) and Pns12-enconding genes were also effective targets for engineering hpRNA-mediated resistance to RDV [35,36,37]. Moreover, transgenic rice materials resistant to RBSDV have been successfully generated by targeting the P9-1-encoding gene using an hpRNA transgene [30]. However, it has been suggested that high-level resistance to multi-partite viruses, which have multiple genomic segments, is relatively more difficult to achieve than monopartite viruses using RNA silencing technologies, such as hpRNA transgenes, because genomic segments that are not targeted could support the replication of the residual targeted RNA segment, preventing its complete destruction [38]. Simultaneously targeting multiple genomic segments using hpRNA transgenes could therefore result in more robust virus resistance. So far, almost all antiviral rice materials generated using the hpRNA technology involved the targeting of only one virus gene [23,30].

This study attempts to create rice germplasm with a stable and durable resistance to RBSDV using the hpRNA technology. As RBSDV is a multipartite virus consisting of 10 genomic segments, we developed an hpRNA construct targeting four genomic segments (*S1*, *S2*, *S6* and *S10*) encoding four important proteins, including RNA polymerase (P1), putative core protein (P2), RNA silencing suppressor (P6) and coat protein (P10). We expected that targeting these proteins would both inhibit viral replication and enhance the host antiviral silencing, resulting in robust and durable viral resistance. Indeed, transgenic rice plants expressing this multi-target hpRNA showed high and durable resistance to RBSDV. Our results demonstrate the feasibility of simultaneously targeting multiple viral genes using hpRNA transgenes for achieving high levels of virus resistance in rice and perhaps other plant species.

## 2. Results

### 2.1. Rice Plants Transformed with an hpRNA Construct Targeting Four RBSDV Genes Show Strong Resistance to RBSDV

We generated rice plants containing an hpRNA construct designed to target four genes, encoding P1 (RNA-dependent RNA polymerase), P2 (a putative core protein), P6 (RNA silencing suppressor) and P10 (major outer capsid protein) of RBSDV, which play an important role in the pathogenicity and multiplication of the virus (Figure S1). The hpRNA construct was transformed into the RBSDV susceptible rice variety Kitaake, and nine positive transgenic rice lines were obtained and confirmed by PCR specific for the *AtFAD2* intron (Figure S2). The primary (T_0_) transgenic plants and their T_1_, T_2_ and T_3_ progeny showed no visible difference to the wild-type Kitaake plants in growth period, plant height and other traits, such as number of tillers and grain yield.

The hpRNA transgenic lines were assayed for RBSDV resistance using T_1_, T_2_ and T_3_ populations. We first investigated the disease incidence rate in the natural field nursery. The efficiency of RBSDV infection was relatively low in the natural field, with only ~11%–22% plants of the susceptible variety Huaidao 5 showing disease symptoms in 2012 and 2013 (Tables S1 and S2). Despite this low efficiency, a clear reduction in disease incidence could be observed for the transgenic lines, particularly for Lines #2, #3 and #4, which showed a ~0%–6% disease rate in these two years (Tables S1 and S2). A greater difference was observed in 2014: the wild-type plants displayed a ~41.44% disease incidence rate, whereas the transgenic lines showed only a ~0%–14% disease rate (Table S3). These results suggested that the transgenic rice lines have enhanced RBSDV resistance.

**Figure 1:**
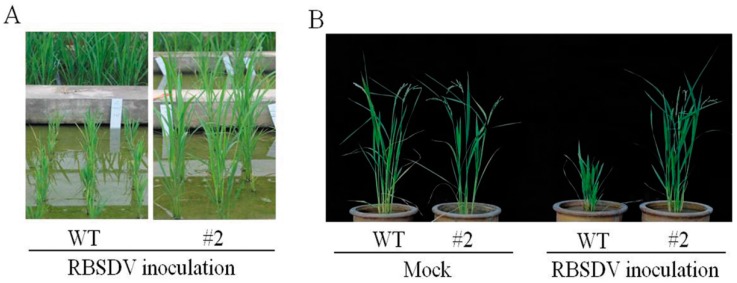
Resistance phenotypes of the transgenic rice plants. Representative plants at 30 days post-inoculation (dpi) (**A**) and 60 dpi (**B**). WT, wild-type Kitaake; #2, hpRNA transgenic rice plant; mock, mock inoculation (negative control).

As the natural field assay was not ideal, we assayed the T_3_ population of the transgenic lines for RBSDV resistance using the artificial inoculation approach that we recently established [39,40,41]. Of the eight T_3_ transgenic populations analysed, five showed strongly-enhanced resistance to RBSDV, with an average disease incidence rate of 0%–12.82%, in contrast to a 100% disease rate for the wild-type plants (Figure 1; Table 1). These results further confirmed the field assay results and indicated that the hpRNA transgene targeting four viral genes is effective at conferring high-level resistance to RBSDV.

### 2.2. RBSDV Accumulation Is Inhibited in the hpRNA Transgenic Plants

**Figure 2:**
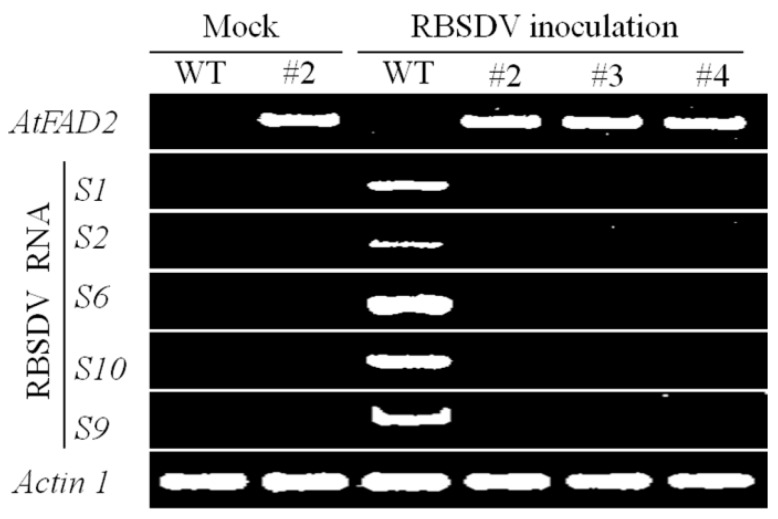
Expression analysis of the hpRNA construct and RBSDV target genes in transgenic rice plants. WT, wild-type Kitaake; #2, #3 and #4, three independent transgenic rice lines; *Actin 1*, internal reference gene; mock, mock inoculation (negative control).

Next, we analysed the expression of the hpRNA transgene and the accumulation of viral RNA in the transgenic rice lines that showed an enhanced RBSDV resistance. It was previously shown that transcript derived from hpRNA transgenes is not fully processed into small RNAs, and therefore, the presence of unprocessed hpRNA can be used to indicate the expression of the hpRNA transgene [42]. The expression of the hpRNA in the transgenic rice lines was confirmed by semi-quantitative RT-PCR using primers designed for the intron spacer of the RNAi vector (Figure 2). As expected, the *S1*, *S2*, *S6*, *S10* and *S9* viral RNAs were detectable in the wild-type Kitaake (Figure 2), indicating successful infection of RBSDV. In contrast, neither the hpRNA target viral RNAs (*S1*, *S2*, *S6*, *S10*), nor the non-target *S9* RNA were detected in the three transgenic rice lines showing both hpRNA expression and a high level of RBSDV resistance after inoculation with SBPH carrying RBSDV (Figure 2). These results indicated that the hpRNA transgene was highly effective at inducing RNA silencing against the viral RNAs, inhibiting viral RNA accumulation and, hence, RBSDV infection and multiplication.

### 2.3. RBSDV Resistance Phenotype Is Linked to the hpRNA Transgene

**Figure 3:**
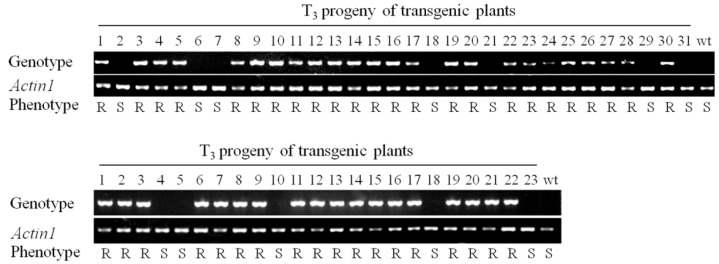
Co-segregation analysis of RBSDV resistance and the presence of the hpRNA transgene in two transgenic rice lines. The presence of the transgene was investigated using primers specific for the *AtFAD2* intron existing as the spacer in the hpRNA transgene. In total, 31 individual plants from Line #2 (**upper**) and 23 individual plants from Line #3 (**lower**) were analysed. *Actin1* was used as a reference for DNA quality. Wild-type plant was used as the negative control of the transgene. The RBSDV resistance (*R*) or susceptible (*S*) phenotypes were based on results from the artificial inoculation experiment.

The small number of RBSDV-susceptible transgenic plants observed in the artificial inoculation assay could be due to the absence of the hpRNA transgene in these individual progeny plants of the segregating transgenic populations. To examine this, we performed PCR genotyping of the RBSDV-resistant and susceptible T_3_ progeny of two transgenic lines (#2 and #3) for the presence of the hpRNA transgene. Of the 31 progeny of Line #2 genotyped, 24 and seven showed presence and absence of the hpRNA transgene sequence, respectively (Figure 3). For the 23 progeny of Line #3 tested, 18 and five showed the presence and absence of the transgene sequence, respectively (Figure 3). Importantly, all T_3_ progeny that contained the hpRNA transgene were resistant to RBSDV, whereas all T_3_ plants without the hpRNA transgene behaved like the wild-type plants and were susceptible to RBSDV (Figure 3). Thus, RBSDV resistance was co-segregating with the hpRNA transgene, indicating that the virus resistance was due to hpRNA-induced RNA silencing.

### 2.4. Abundant hpRNA-Derived siRNAs Are Detected in Transgenic Rice Using Small RNA Deep Sequencing

To confirm the efficacy of the hpRNA transgene, we examined the accumulation of hpRNA transgene-derived siRNAs in the RBSDV-resistant transgenic Lines #2, #3 and #4 and the wild-type Kitaake plants using Illumina deep sequencing. In total, 1.84, 1.73, 1.65 and 1.86 million unique small RNA reads were generated for the transgenic Lines #2, #3, #4 and the wild-type plant, respectively. In all samples, the 24-nt siRNAs were the most abundant, accounting for ~40%–44% of the total unique small RNA reads (Figure S3). This is consistent with the normal sRNA size distribution in plants such as rice and *Arabidopsis* [43,44], indicating the good quality of the sequencing data.

**Figure 4:**
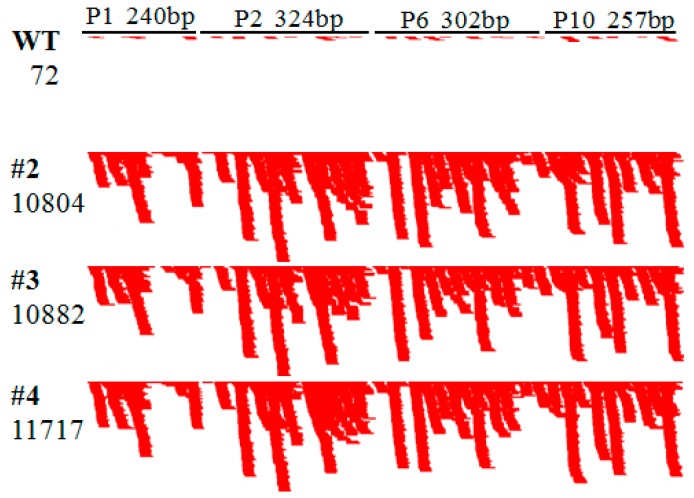
Distribution of the hpRNA-derived siRNAs across the four sequence fragments of the fusion viral sequence from the genes encoding P1, P2, P6 and P10. WT, wild-type Kitaake; #2, #3 and #4, three independent transgenic rice lines resistant to RBSDV.

We mapped the unique small RNA reads to the fusion viral sequence in the hpRNA transgene and detected 10,804, 10,882 and 11,617 unique small RNAs from Lines #2, #3, and #4 that could be mapped, respectively (Figure 4). Interestingly, 72 mapped unique small RNAs, from a total of 1.86 million reads, were also detected in the wild-type plant, which could imply the integration of RBSDV-like sequences in the rice genome as reported for the non-retroviral RNA virus elements recently discovered in both plants and animals [45,46]. However, compared to the transgenic lines, the number of hpRNA-matching small RNAs in the wild-type plant is almost negligible. The deep sequencing data confirmed that the hpRNA was expressed and efficiently processed by Dicer-like proteins into siRNAs in the transgenic plants, inducing antiviral silencing against RBSDV.

**Figure 5:**
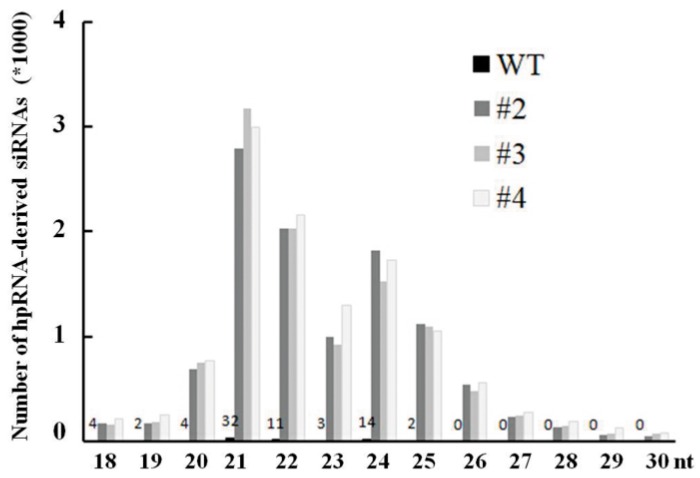
Size distribution of the hpRNA-derived siRNAs. WT, wild-type Kitaake; #2, #3 and #4, three independent transgenic rice lines resistant to RBSDV.

While the 24-nt size class was the most abundant for the total small RNA population, 21- and 22-nt small RNAs were the dominant size classes (25.7%–29.2%) for the hpRNA-derived siRNAs (Figure 5), which is consistent with previous reports showing that hpRNA is processed primarily by DCL4 (21 nt) and DCL2 [33]. Interestingly, the P1 sequence, near the “open end” of the predicted hpRNA stem-loop structure, was associated with less abundant siRNAs than the other three sequences of the fusion fragment in the hpRNA transgene (Figure 4).

## 3. Discussion

In recent years, rapid development in plant biotechnology has provided a diverse variety of feasible methods for generating antiviral rice germplasm [21]. Previous reports have demonstrated that virus resistance in rice can be achieved by silencing one viral gene using RNA silencing-based technology. For example, transgenic rice plants containing an hpRNA transgene targeting the P9-1-encoding gene were almost immune to RBSDV infection [30]. Rice plants expressing hpRNA targeting the nucleocapsid protein *pC5* gene or the movement protein *pC6* gene of the rice grassy stunt virus (RGSV) showed resistance against RGSV infection [47]. However, no study has been reported on achieving virus resistance by simultaneously silencing multiple viral genes, which is believed to be more effective than targeting a single viral gene at conferring resistance against multipartite viruses [38]. In this study, we generated transgenic rice lines expressing an hpRNA transgene targeting four RBSDV genes encoding proteins of multiple functions essential for the replication, encapsidation and spread of the virus and for counter-defence against host antiviral RNA silencing, including the RNA-dependent RNA polymerase, putative core protein, RNA silencing suppressor and coat protein. We expected that, by targeting multiple genes from multiple genomic segments of RBSDV, high levels of virus resistance could be achieved. Indeed, transgenic rice plants expressing the RBSDV hpRNA showed strong virus resistance in both the field and artificial assay systems. Whether or not a similar resistance level can be achieved by targeting one or two of the four genes used in this study requires further investigation. Nevertheless, we believe that more durable and stable resistance could be better achieved by targeting multiple genes and genomic fragments for the multipartite virus RBSDV.

RT-PCR analyses showed that all four target viral RNAs were efficiently suppressed in the transgenic rice plants with strong resistance to RBSDV, suggesting that the four viral gene fragments in the hpRNA are all processed efficiently by Dicer-like enzymes into siRNAs. This was confirmed by the small RNA deep sequencing analysis, which showed that abundant siRNAs were detectable from all four sequences of the hpRNA. hpRNA in plants is processed primarily by DCL4 and DCL2 into 21- and 22-nt siRNAs [33,48,49]. Consistent with this, the 21- and 22-nt size classes of siRNAs are the predominant population of the hpRNA-derived siRNAs in the transgenic rice plants, in contrast to the overall small RNA population, which is dominated by the 24-nt size class. All three transgenic lines analysed showed a similar abundance and size distribution pattern of siRNA, suggesting that the maize ubiquitin promoter conferred a high and consistent level of expression of the hpRNA transgene in the independent transgenic lines. 

It is difficult to evaluate the resistance level of RBSDV in rice by using the method of natural field nursery, because of the difficulties in controlling the density and percentage of the insect vector SBPH carrying RBSDV [50]. In our field RBSDV nursery experiments, the disease incidence rate was quite low for the RBSDV susceptible wild-type Kitaake and Huaidao 5, particularly during the 2012 and 2013 seasons, probably due to a relatively low density and percentage of SBPH carrying RBSDV during the time of the experiments. The disease rate for the wild-type plants was significantly higher in 2014, suggesting that a reliable result could be achieved by multiple years’ experiments in the field. The absence of an efficient system for the evaluation of RBSDV resistance has restricted the ability to uncover resistance resources, limited the development of resistance breeding intermediates and hindered genetic studies on RBSDV in rice [40]. Recently, we successfully established an artificial inoculation approach for reliable evaluation of RBSDV resistance under controlled conditions [39,40,41]. In the present study, we used this artificial inoculation approach to investigate RBSDV resistance levels of the hpRNA transgenic rice plants and obtained reliable results.

## 4. Materials and Methods

### 4.1. Construction and Transformation of RNAi Vector

We first amplified the individual gene fragments (215, 299, 277 and 257 bp for P1, P2, P6 and P10, respectively) separately based on the sequence information from NCBI (Tables S4 and S5; Figure S1A) and then assembled them to form a single fusion fragment (1125 bp) using overlapping PCR [51] (Figure S1B,C). This chimeric segment was then placed in the sense and antisense orientations separated by an intron (from *FAD2* of *Arabidopsis*) to form the hpRNA construct (Figure S1D,E). The intron serves both as a spacer for easy cloning and to enhance silencing efficiency [52]. This sense-intron-antisense fragment was cloned between the maize ubiquitin (Ubi) promoter and the nopaline synthase (Nos) terminator, and this expression cassette then was inserted into the binary expression vector pWBVec8 containing hygromycin resistance gene as the selective marker [53]. The construct was transformed into *Agrobacterium tumefaciens* strain EHA105 using a freeze-thaw method. Transformation of rice (*Oryza sativa Japonica* variety Kitaake) was performed following the *Agrobacterium*-mediated transformation method using calli derived from mature seed and hygromycin B as the selective agent [54].

### 4.2. Plant Materials and Growth Conditions

Primary transgenic rice plants (T_0_) were grown in a growth chamber (12-h photoperiod; 28 °C; 70% relative humidity; light strength, 30,000 lx) with slow-release fertilizer added to the soil. Once recovered, transgenic plants were transplanted into bigger pots under field growth conditions for sample collection and seed harvesting. The presence of the hpRNA transgene in the T_1_ or T_2_ generation was confirmed by PCR, and positive transgenic plants were selected for producing subsequent generation. Plants of each generation were self-pollinated. For analysis of RBSDV resistance and for other molecular analyses, all plants (transgenic rice and wild-type Kitaake) were grown in a paddy field under natural light and temperature conditions.

### 4.3. Analysis of RBSDV Resistance

Two methods, field RBSDV nursery and artificial inoculation of SBPH carrying RBSDV, were used to analyse the RBSDV resistance level of transgenic rice.

#### 4.3.1. Disease Assay under the Field RBSDV Nursery Conditions

Transgenic rice plants and the RBSDV susceptible wild-type Kitaake were planted in the paddy field RBSDV nursery with the presence of viruliferous SBPH in the summer of 2012, 2013 and 2014. During the period of rice growth and development, no pesticides and antivirus agents were applied to the plants. The number of plants resistant or susceptible to RBSDV was recorded, and the disease incidence rate was calculated at the tillering stage [30,39,40].

#### 4.3.2. Disease Assay Using the Artificial Inoculation Method

In order to ensure the reliability of disease assay, we assayed the transgenic plants for RBSDV resistance using an artificial inoculation approach. SBPH were first fed on viruliferous rice cultivar Huaidao 5, and the SBPH carrying RBSDV were then moved to the beakers with Wuyujing 3 seedlings until through the 12-day latent period. The reverse transcription-loop mediated isothermal amplification (RT-LAMP) approach was used to detect RBSDV in SBPH [39], and the percentage of viruliferous SBPH was about 30%–40%. Ten-day-old rice seedlings were inoculated with a density of 3–4 viruliferous SBPH per plant in a cage. Virus-free SBPH was used for mock inoculation (negative control). After 3 days, SBPH were removed by spraying with insecticide. Subsequently, SBPH-fed plants were transplanted into an insect-free greenhouse at 25 ± 3 °C under natural sunlight for evaluation of disease symptoms. The total number of diseased plants in every transgenic rice line is recorded at the tillering stage, and the disease incidence rate was calculated [40,41].

### 4.4. RT-PCR

Leaf samples from transgenic rice lines and wild-type Kitaake were collected for total RNA extraction. Total RNA was extracted using the TRIzol Kit according to the instruction manual (TaKaRa, Dalian, China) and treated with RNase-free DNase I (Promega, Madison, WI, USA). cDNA was synthesized using PrimeScript™ reverse transcriptase from total RNA according to the manufacturer’s instructions (TaKaRa, Dalian, China). The primers for detecting *S1*, *S2*, *S6* and *S10* RNA of RBSDV were designed to avoid amplification of the hpRNA fragments from transgenic plants (Table S4). *Actin 1* was used as the internal reference gene for normalization of RNA quantities.

### 4.5. Deep Sequencing Analysis of hpRNA-Derived siRNA

Leaves of three independent transgenic lines (#2, #3 and #4) and wild-type Kitaake were collected at the tillering stage and used for total RNA extraction using the TRIzol Kit according to the manufacturer’s instructions (TaKaRa, Dalian, China). Small RNA sequencing libraries were prepared from the total RNA samples using the TruSeq Small RNA Sample Preparation Kit (Illumina Inc., San Diego, CA, USA) and sequenced using the Illumina Genome Analyzer II (Illumina Inc., San Diego, CA, USA). The CLC Genomics Workbench5 software was used for analysing the small RNA sequence data.

## 5. Conclusion

In this study, we successfully generated transgenic rice lines with high-level resistance to RBSDV by targeting simultaneously four viral genes using the hpRNA transgene technology. Our results showed that hpRNA transgenes targeting multiple viral genes can be effectively used to generate RBSDV-resistant rice germplasm, which can provide alternative genetic resources for breeding RBSDV resistance. It can be anticipated that the same approach can be applied to engineering resistance to other viruses in other plant systems.

## Figures and Tables

**Table 1:** Disease assay of the T_3_ transgenic rice plants by the artificial inoculation method.

Rice Materials		Disease Response of the Tested Plants			
		*N* ^a^	*S* ^b^	*R* ^c^	Incidence Rate (%)
Transgenic lines	#1	39	5 ^d^	34	12.82
	#2	37	0	37	0.00
	#3	38	2 ^d^	36	5.26
	#4	36	1 ^d^	35	2.78
	#5	35	4 ^d^	31	11.43
Wild-type		40	40	0	100.00
Huaidao 5 ^e^		38	38	0	100.00

^a^ Total number of rice plants examined; ^b^ typical symptoms were observed at 30 days post-inoculation; ^c^ no symptoms were observed during the whole growth period; ^d^ the intron of *AtFAD2* was not detected in these plants by PCR; ^e^ Huaidao 5 is an RBSDV-susceptible *Japonica* variety in Jiangsu Province of China, used as negative controls.

## Supplementary Materials

Supplementary materials can be found at http://www.mdpi.com/1422-0067/17/5/705/s1.
