# Supplementary material for: Hairpin RNA Targeting Multiple Viral Genes Confers Strong Resistance to Rice Black-Streaked Dwarf Virus
Source: Int J Mol Sci. 2016 May 11;17(5):705. doi: 10.3390/ijms17050705 (PMC4881527; doi:10.3390/ijms17050705)
Supplement: Supplementary file 1 [file ijms-17-00705-s001.pdf]

Supplementary Materials: Hairpin RNA Targeting Multiple Viral Genes Confers Strong Resistance to Rice Black-Streaked Dwarf Virus

Fangquan Wang, Wenqi Li, Jinyan Zhu, Fangjun Fan, Jun Wang, Weigong Zhong, Ming-Bo Wang, Qing Liu, Qian-Hao Zhu, Tong Zhou, Ying Lan, Yijun Zhou and Jie Yang

Table S1. Disease assay of transgenic rice in the field RBSDV nursery (2012).

Rice Materials		Disease Response of the Tested Plants			
		N [a]	*S* [b]	*R* [c]	Incidence Rate (%)
**Repeat 1**					
T_1 transgenic lines	#1	39	4 [d]	35	10.26
	#2	35	0	35	0.00
	#3	36	1 [d]	35	2.78
	#4	37	0	37	0.00
	#5	28	0	28	0.00
	#6	38	10 [d]	28	26.32
	#7	34	6 [d]	28	17.65
	#8	37	2 [d]	35	5.41
Wild-type		34	10	24	29.41
Huaidao 5 [e]		32	7	25	21.88
**Repeat 2**					
**Rice Materials**		**Disease Response of the Tested Plants**			
		N [a]	*S* [b]	*R* [c]	Incidence Rate (%)
T_1 transgenic lines	#1	38	2 [d]	36	5.26
	#2	34	0	34	0.00
	#3	35	0	35	0.00
	#4	39	2 [d]	37	5.13
	#5	37	4 [d]	33	10.81
	#6	36	7 [d]	29	19.44
	#7	37	5 [d]	32	13.51
	#8	39	6 [d]	33	15.38
Wild-type		37	4	33	10.81
Huaidao 5 [e]		36	4	32	11.11

[a] Total number of rice plants examined; [b] typical disease symptoms were observed at 30 days post-inoculation; [c] no symptoms were observed during the whole growth period; [d] the intron of *AtFAD2* was not detected in these plants by PCR; [e] Huaidao 5 is a susceptible *Japonica* variety in Jiangsu Province of China used as the negative control.

Table S2. Disease assay of transgenic rice in the field RBSDV nursery (2013).

Repeat 1					
**Rice Materials**		**Disease Response of the Tested Plants**			
		N [a]	***S*** [b]	***R*** [c]	**Incidence Rate (%)**
T_2 transgenic lines	#1	34	0	34	0.00
	#2	37	0	37	0.00
	#3	39	1 [d]	38	2.56
	#4	40	1 [d]	39	2.50
	#5	38	4 [d]	34	10.53
	#7	38	3 [d]	35	17.5
	#8	36	9 [d]	27	25.00
Wild-type		34	6	28	17.65
Huaidao 5 [e]		39	5	34	12.82
**Repeat 2**					
**Rice Materials**		**Disease Response of the Tested Plants**			
		N [a]	***S*** [b]	***R*** [c]	**Incidence Rate (%)**
T_2 transgenic lines	#1	34	2 [d]	32	5.88
	#2	38	0	38	0.00
	#3	35	0	35	0.00
	#4	37	0	37	0.00
	#5	37	3 [d]	34	8.11
	#7	40	7 [d]	33	17.5
	#8	39	3 [d]	36	7.92
Wild-type		38	5	33	13.16
Huaidao 5 [e]		36	8	28	22.22

[a] Total number of rice plants examined; [b] typical disease symptoms were observed at 30 days post-inoculation; [c] no symptoms were observed during the whole growth period; [d] the intron of *AtFAD2* was not detected in these plants by PCR; [e] Huaidao 5 is a susceptible *Japonica* variety in Jiangsu Province of China used as the negative control.

Table S3. Disease assay of transgenic rice in the field RBSDV nursery (2014).

**Rice Materials**		**Disease Response of the Tested Plants**			
		N [a]	***S*** [b]	***R*** [c]	**Incidence Rate (%)**
T_3 transgenic lines	#1	86	4 [d]	82	4.65
	#2	103	0	103	0
	#3	64	9 [d]	55	14.06
	#4	135	4 [d]	131	2.96
	#5	50	1 [d]	49	2.00
	#6	160	0	160	0
	#7	74	2 [d]	72	2.70
	#8	149	0	149	0
Wild-type		111	46	65	41.44

[a] Total number of rice plants examined; [b] typical disease symptoms were observed at 30 days post-inoculation; [c] no symptoms were observed during the whole growth period; [d] the intron of *AtFAD2* was not detected in these plants by PCR.

Table S4. Primers used in this study.

Primer	Sequence * (5′–3′)	Genes	Accession Numbers
	**Primers for the RNAi vector construct**		
S1-For	GGTGGAACGAAAGTTCAGTAGATC	*S1*	AJ294757
S1-Rev	GGTGCTTCAGGCAAAAAGTTGTCAGAATTTGGACTACACTTGGACGAA		
S2-For	CTGACAACTTTTTGCCTGAAGCACCAGAGCGACAAGAAGAATCGAAA	*S2*	AJ409145
S2-Rev	TGGGATCAGACGAAAATATTGGACGCAAAAGTAGTTGTGTAAGCGGG		
S6-For	CGTCCAATATTTTCGTCTGATCCCACTCGAATCATCCGTCACTTCTGAGT	*S6*	AJ409148
S6-Rev	TGCGTTTGTTGACCATTACCATGAAGGACAAAACCTTTCCAATTATCGAG		
S10-For	TTCATGGTAATGGTCAACAAACGCAGGAAACATTACTTTGAAGCCC	*S10*	AF459813
S10-Rev	CCACCATAATGTGTAACATCCGTA		
iS1-For	GATGGATCCGGTGGAACGAAAGTTCAGTAGATC (*Bam*HI)		
iS10-Rev	GTAGCGGCCGCCCACCATAATGTGTAACATCCGTA (*Not*I)		
iS10-For	GATCTCGAGCCACCATAATGTGTAACATCCGTA (*Xho*I)		
iS1-Rev	GATGAATTCGGTGGAACGAAAGTTCAGTAGATC (*Eco*RI)		
	**Primers for gene expression**		
FAD-For	GAATTTCTCCGCTCACGA	*AtFAD2*	AJ271841
FAD-Rev	ATTTCCACCAACCCACCA		
S1-exp-For	CCTATGTTAACTTTCGCCAT	*S1*	AJ294757
S1-exp-Rev	TTCAATTACTCCATCACGTT		
S2-exp-For	CAGATTATCCGTTTATTAGCTC	*S2*	AJ409145
S2-exp-Rev	TTTCTTGTCCATCAAACGGTA		
S6-exp-For	TGCACACTTATCAATCACGTT	*S6*	AJ409148
S6-exp-Rev	GATACCTGTAGTTGACCGTTC		
S10-exp-For	TGCCACTAATACTTCAACCAC	*S10*	AF459813
S10-exp-Rev	GCACAACACTGAACTAGTCG		
S9-For	ATGGCAGACCAAGAGCGGAG	*S9*	NC_003731
S9-Rev	GGCATTTCAACTATTTTCTTCTC		
ACT-For	AGCCACACTGTCCCCATCTA	*Actin 1*	NM_001057621
ACT-Rev	AGCAAGGTCGAGACGAAGGA		

* Restriction sites/recombination sites are underlined.

Table S5. Sequence information of the four RBSDV target genes (*S1*, *S2*, *S6* and *S10*) used in his study.

Target Gene	Length (bp)	ORF Position	Protein Size (kDa)	Functions and Properties	RT-PCR Locus	Reference
S1	4501	36–4427	1464 (168.8)	RNA-dependent RNA polymerase	38–252	[9]
S2	3812	46–3723	1226 (141.5)	Core protein	117–415	[9]
S6	2645	82–2457	792 (89.9)	Silencing suppressor	202–478	[9]
S10	1801	22–1695	558 (63.3)	Coat protein	472–728	[11]

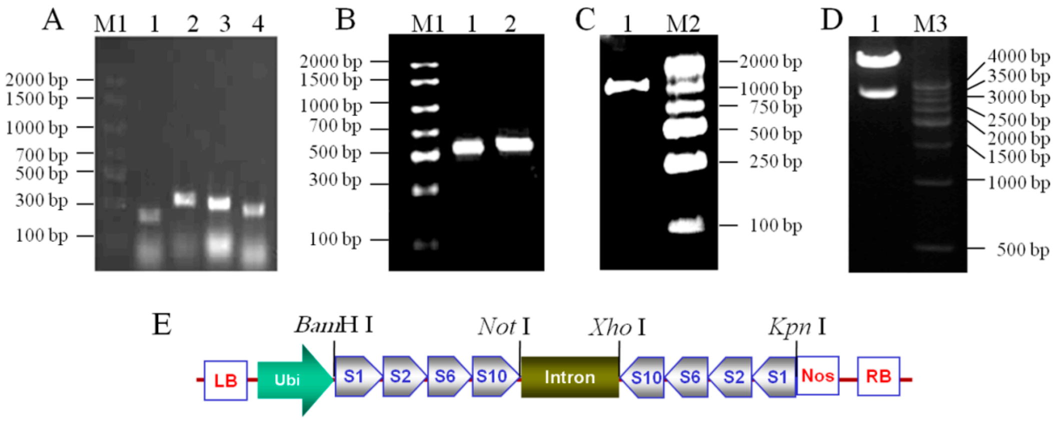

Figure S1. Construction of the hpRNA transformation vector. (**A**) Amplification of the individual gene fragments from the four target genes of RBSDV. Lanes 1–4 are the fragments of the *S1*, *S2*, *S6* and *S10* gene, respectively; (**B**) Concatenation of two segments. Lanes 1 and 2 show the concatenated intermediates of the *S1* + *S2* and the *S6* + *S10* fragments, respectively; (**C**) Concatenation of four segments. Lane 1 is the full-length fusion fragment; (**D**) Verification of the hpRNA vector. Lane 1 is the *Bam*HI/*Kpn*I double-digestion product containing the full-length fusion fragment and the vector backbone; (**E**) The schematic diagram shows the composition and organization of the hpRNA construct. LB and RB, left border and right border of the T-DNA, respectively. Ubi, maize ubiquitin promoter. Intron, the intron from *AtFAD2* serving as the spacer separating the inverted repeats of the viral sequence. Nos, the nopaline synthase terminator. M1, DNA Marker IV; M2, DNA Marker DL 2000; M3, 500-bp DNA Ladder.

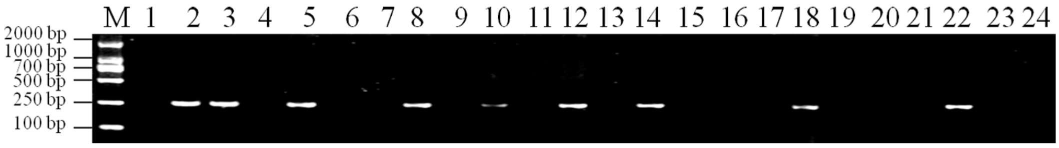

Figure S2. Identification of positive transgenic rice plants using PCR specific for the intron of *AtFAD2*. Lanes 1–21, the amplification results of 21 different transgenic rice plants; Lane 22, positive control; Lane 23, untransformed plant (negative control); Lane 24, water (negative control); M, DNA Marker DL 2000.

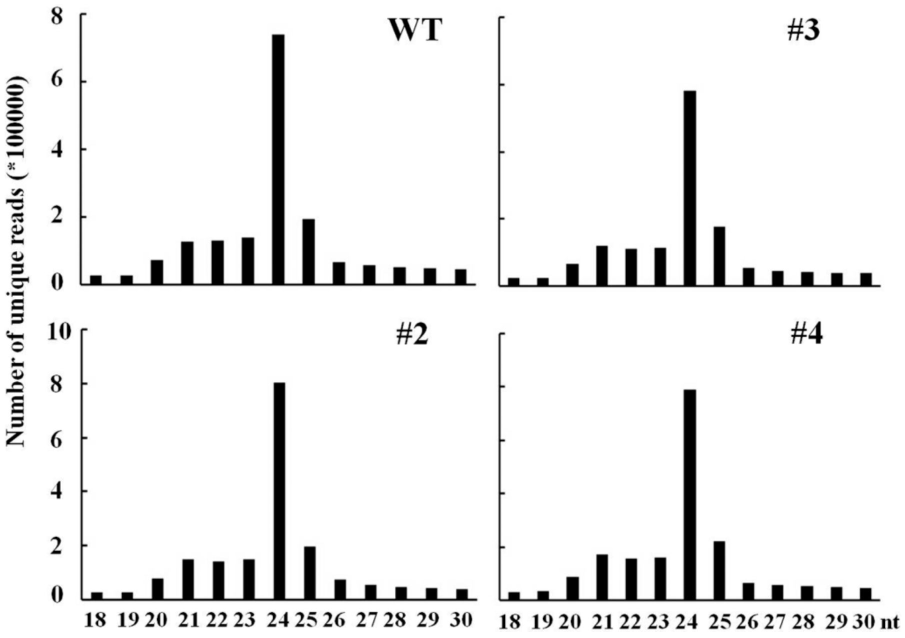

Figure S3. Size distribution of unique small RNAs from the three transgenic rice lines. WT, wild-type Kitaake; #2, #3 and #4, three independent transgenic rice lines resistant to RBSDV.
